# The Road Not So Travelled: Should Measurement of Vitamin D Epimers during Pregnancy Affect Our Clinical Decisions?

**DOI:** 10.3390/nu9020090

**Published:** 2017-01-28

**Authors:** Spyridon N. Karras, Kalliopi Kotsa, Elena Angeloudi, Pantelis Zebekakis, Declan P. Naughton

**Affiliations:** 1 Division of Endocrinology and Metabolism, First Department of Internal Medicine, Medical School, Aristotle University of Thessaloniki, AHEPA Hospital, 55535 Thessaloniki, Greece; kalmanthou@yahoo.gr (K.K.); elena-angeloudi@hotmail.com (E.A.); pzempeka@auth.gr (P.Z.); 2 School of Life Sciences, Pharmacy and Chemistry, Kingston University London, Penrhyn Road, Kingston upon Thames, Surrey KT1 2EE, UK; D.Naughton@kingston.ac.uk

**Keywords:** vitamin D, epimers, pregnancy

## Abstract

Observational studies suggest an adverse effect of maternal hypovitaminosis D during pregnancy. However, intervention studies failed to show convincing benefit from vitamin D supplementation during pregnancy. With analytical advances, vitamin D can now be measured in ten forms—including as epimers—which were thought to be biologically inactive, but can critically impair immunoassays. The aim of this commentary is to highlight the potential clinical and analytical significance of vitamin D epimers in the interpretation of vitamin D roles in pregnancy. Epimers may contribute a considerable proportion of total vitamin D—especially in the neonate—which renders the majority of common assays questionable. Furthermore, epimers have been suggested to have activity in laboratory studies, and evidence suggests that the fetus contributes significantly to epimer production. Maternal epimer levels contribute significantly to predict neonate circulating 25-hydroxyvitamin D concentrations. In conclusion, the existence of various vitamin D forms (such as epimers) has been established, and their clinical significance remains obscure. These results underscore the need for accurate measurements to appraise vitamin D status, in order to understand the current gap between observational and supplementation studies on the field.

## 1. Introduction

In the past years, vitamin D has gained in clinical significance, since its presence has been associated with multiple health outcomes, mostly with the maintenance of bone preservation across the lifespan [1]. This significance is also highlighted by the fact that hypovitaminosis D has been hypothesized to be involved in the pathogenesis of several diseases, although improvement in bone outcomes remain the only confirmed health outcome [1,2]. Gradually, intervention studies have been conducted, focusing on the potential beneficial effects of vitamin D supplementation on these outcomes [3,4,5,6,7].

However, most of these studies failed to show a benefit from vitamin D supplementation during pregnancy, in several outcomes, implying that there is a knowledge gap between observational and supplementation studies on the field [8,9,10]. The enzymatic machinery of the cell, 25-hydroxyvitamin D-1 α-hydroxylase, acts on its primary target—25-hydroxyvitamin D (25(OH)D)—to produce the active form of vitamin D, the 1,25(OH)2D, which in turn interacts with the nuclear vitamin D receptor (VDR) [2]. The above reaction is the main metabolic pathway of vitamin D, but there are also several minor metabolic pathways on the road to the final and prevailing vitamin D metabolites. In this regard, vitamin D epimers result from the epimerization pathway of the plethora of metabolites of vitamin D3 [11]. The biological role of those epimers has yet to be elucidated, as a result of their potential impact on serum assays resulting in analytical and perhaps clinical issues of vitamin D status interpretation and supplementation [8] in the general population [9]. Recently, new emerging theories [8,9,10] highlight the need to further investigate the analytical and clinical significance of epimers during pregnancy, since under specific conditions there is a potent correlation between those compounds and the health status of an ongoing pregnancy, as well as foetal birth parameters or potential adverse outcomes [12,13].

The aim of this commentary is to highlight the potential clinical and analytical significance of vitamin D epimers in the interpretation of maternal vitamin D status. It will be based on results focused on the physiological and pathophysiological aspects of the roles of the epimer forms during pregnancy, in the context of the current gap between observational and supplementation studies on the field.

## 2. Physiological role of vitamin D epimers 

### 2.1. Overview of Epimer Physiology: Non-Pregnant State

In order to become active, 25(OH)D requires hydroxylation that takes place mainly in the kidney. This results in the conversion to the active form 1,25(OH)2D, or to 24R,25-dihydroxyvitaminD3, the presumably inactive form [14]. Except for those primary metabolites of vitamin D, focus has recently been brought on minor intermediates of the catabolic process of vitamin D. 

1,25(OH)2D3 is subjected to enzymatic C-24/C-23 hydroxylation [15]. The C-24 oxidation pathway leads to the conversion of 1,25(OH)2D3 into calcitroic acid, and the C-23 oxidation pathway leads to the formation of 1,25(OH)2D3-26,23-lactone. Whereas this oxidation is significantly associated with inactivation, epimerization has been linked to the retention of significant biological activity [14,15]. The products of epimerization are called epimers. The C-3 epimerization pathway of 1,25(OH)2D3 produces 3-epi-1,25(OH)2D3, and the two molecules only differ in the configuration of the hydroxyl group at the 3 carbon position. 

Likewise, 3-epi-25(OH)D3 was then identified as C-3 epimerized metabolites of 25(OH)D, and 3-epi-24,25(OH)2D3 and 3-epi-24,25(OH)2D3-24-glucuronide were identified as C-3 epimerized metabolites of 24,25(OH)2D3 [12,13,14,15]. After all, 25(OH)D3 is the most abundant vitamin D metabolite in circulation, and 3-epi-25(OH)D3 is the most prevalent epimer of 25(OH)D [14,15]. Epimers need to be separated during the measurement of true vitamin D status, because they can display compatible chromatography with vitamin D metabolites [14,15,16,17].

There is adequate evidence that at least one epimer form of vitamin D is present in adult blood in significant circulating concentrations, although a small number of studies demonstrate contradicting evidence [18,19,20,21]. Reasons for this controversy are likely due to differences in high-throughput liquid chromatography-tandem mass spectrometry (LC-MS/MS) techniques in the quantification of the epimers between studies, as well as to the different study populations. The formation of the epimer has been reported in several cell lines, suggesting that they can be formed endogenously [20], which was further supported by tracking epimer levels in an additional study [19]. 

Considering the above information, and given the considerable growth in interest in the roles of epimer forms of vitamin D in vivo [11,16], it is essential to selectively and accurately measure multiple forms of vitamin D separately [15,16,18]. The development of rapid accurate assays with this capability is certainly a tantalizing prospect. Within the past five years, a number of research laboratories have developed or acquired the ability to conduct advanced LC-MS/MS analyses (high-throughput liquid chromatography-tandem mass spectrometry), affording the capability to measure up to ten analogues of vitamin D in a short (>10 min) run. This is a technique that combines the physical separation capabilities of liquid chromatography (or HPLC) with the mass analysis capabilities of mass spectrometry (MS). Using improved LC-MS/MS methods, the C-3 epimer of 25(OH)D was measured at levels between 5 and 92 ng/mL in 22.2% of infants, contributing between 8.7% and 61.1% to the total 25(OH)D [19]. Notably, the proportion of the C-3 epimer appeared to decline in the first year post-partum. However, the same study failed to detect the C-3 epimer in either healthy adult blood samples or in those with compromised liver function. With advances in assay methodology, a subsequent study revealed that the C-3 epimer was detected in adult blood samples at levels between 0.5 and 2.5 ng/mL, which were between 2.6% and 16.7% of the 25(OH)D levels [20]. In addition, in a large adult cohort, concentrations of the epimer were quantifiable (≥1.41 ng/mL) in 33.4% of whites and 15.0% of blacks, and made up on average 3.23% and 2.25% of total D3 (epimer + 25(OH)D3) concentrations, respectively [21]. Low concentrations of the D3 epimer were also present in adult serum, and overall the epimer concentration was moderately correlated with the 25(OH)D3 concentration.

A later LC-MS/MS study reported the C-3 epimer ratio in 214 samples with a mean value of 1.5 ± 1.9 ng/mL (range 0.1–23.7 ng/mL), giving a ratio of C-3 epimer to 25(OH)D3 ranging from 0% to 25.5% [22]. More recently, every sample in a large cohort (*n* = 1148) was shown to contain the C-3 epimer of 25(OH)D3, with a relative abundance (compared to the total 25(OH)D) ranging between 1.8% and 24.8% [23]. This study also found the C-3 epimer of 25(OH)D2 in 88 (7.7%) of the subjects in Thailand. A further study (*n* = 1122) revealed that all samples except two had detectable C-epimer of 23OHD3, and in the quantified samples (96.4%), the levels of epimer were at circa 4.5% of the 25(OH)D [24]. 

### 2.2. Biological Significance of Vitamin D Epimers

Although being supported by a limited number of studies, the physiological role of the epimers has yet to be clarified. The C-3 epimer demonstrated compatible biochemical activity to the active 1,25(OH)2D3 form in cell culture for roles in coactivator peptide recruitment and induction of expression of vitamin D target genes [14]—a phenomenon which implies potential pharmacological roles of the epimer forms in vivo. In detail, Molnar et al. [14] reported significant biological activity and similar biochemical properties of 1α,25(OH)2-3-epi-D3 compared to 1α, 25(OH)2D3 in primary human keratinocytes. In all experiments, highly comparable time courses of incubation of 1 α,25(OH)2D3 and C-3 epimer were recorded with 3-epimer exceeding 1 α,25(OH)2D3 concentration after longer incubation.

Human keratinocytes could provide an ideal cellular model to investigate the anti-proliferative actions of C-3 epimer, since it has been found in a high concentration in this tissue [14]. On that basis, the dose-dependent anti-proliferative effects of 1 α,25(OH)2D3 and 1 α,25(OH)2-3-epiD3 using 3H-thymidine incorporation assay were evaluated. The half maximal inhibitory concentration (IC50) values for 1 α,25(OH)2D3 and C-3 epimer were highly similar (41.4 and 66.1 nM, respectively) with no significant statistical difference, indicating a similar anti-proliferative activity. Recently, exogenous C-3 epimer was as effective as 25(OH)D3 in supporting bone mineral accretion in both male and female rats [25]. Therefore, the significance of epimers in the context of their possible cell-specific effects in the placenta may provide further insights into vitamin D homeostasis during pregnancy [26].

## 3. Vitamin D Epimers during Pregnancy, Birth, and Early and Neonatal Period

Previous research has established the presence of the C-3 epimer at birth and its decrease in the first year after birth [19,27]. In addition, a number of studies [8,28] have applied precise techniques in order to analyze concentrations of multiple forms of vitamin D in pregnancy. Extending the analyses to the maternal–neonate pair, similar levels of epimers of 25(OH)D2 and 25(OH)D3 were found in mother (4.8 ± 7.8 ng/mL) and neonates (4.5 ± 4.7 ng/mL) which contributed approximately 25% of the total circulating vitamin D [11]. Linear regression modelling was used to show which characteristics predict levels of neonate 25(OH)D, and maternal levels of epimers contributed 11.9%, implying a potential role of epimers of both maternal and neonatal vitamin D equilibrium. A later study in maternal–neonate pairs reported similar levels of C-3 epimer for maternal and cord samples (2.4 vs. 2.2 ng/mL) with the proportion of epimer of the total 25(OH)D as 6.5% in maternal and 10.5% in cord samples [28]. 

In both studies, however, advanced LC-MS/MS analyses were used [11,28], allowing accurate quantification of vitamin D isoforms, which in turn resulted in accurate determination of both maternal and neonatal 25(OH)D concentrations in terms of vitamin D insufficiency or deficiency. Specifically, 24.7% and 22.2% of the measured vitamin D forms were for the epimer forms for mothers and neonates, respectively [11]. The same cohort of maternal–neonatal dyads would exhibit higher proportional classification of their vitamin D status if a different assay was used, not allowing the measurement of multiple forms of vitamin D (including epimers) separately.

## 4. What Is the Role of Epimer Measurement in Defining Maternal Hypovitaminosis D?

### 4.1. Analytical Issues

LC-MS/MS is a powerful technique that has very high specificity, making it useful in the discrimination between all forms of vitamin D [20,29].

Many clinical studies rely on immunoassay techniques—which are undergoing constant development—for studies in the general population as well in infants/pregnancy, although the utilization of these techniques in infants can be problematic [30]. Extended efforts for the development of a precise immunoassay for 25(OH)D do exist [31], and it is feasible that these could result in separating multiple metabolites, including epimers. Immunoassays generally do not detect the epimer forms, since cross-reactivity is typically ~1% [15,22,28,29,30]. Results to date point to the need of the widespread adoption of chromatography methods, where the analysis is evidenced by the retention period on the chromatographic column and by measuring several fragmentation events using multiple reaction monitoring [20]. This latter point is critical to deconvolute the roles of all ten forms of vitamin D in pregnancy, in particular cases where vitamin D supplementation is warranted. However, it should be noted that not all LC-MS/MS techniques separate or quantify the epimer forms, since without adequate separation, it may coelute with 25(OH)D [20,29]. 

It should be noted that in order to distinguish the multiple bioactive vitamin D metabolites (including epimers), stringent sample collection and processing procedures may be required, although no such results are available. Moreover, there is a wide heterogeneity in reporting on the sample collection procedures and processing from maternal versus foetal sera for vitamin D epimer analyses by various studies to date [8,9,10,14].

### 4.2. Should Measurement of Epimer Concentration Affect Clinical Decisions? 

Previous findings have suggested that the epimers are inactive, and they were identified under this assumption [15,16,18]. However, even if epimers lack any direct pharmacological function, it is feasible that they play an indirect role by acting as competitors for binding proteins, receptors, or even for hydroxylases required to activate the pro-forms and thus regulating vitamin D homeostasis. This issue has not been addressed in most observational or supplementation studies reported so far, where immunoassays were used for the determination of vitamin D status.

Finally, there is a clear association of detected epimer concentrations with anthropometric and biochemical measurement that must be further unveiled after studies conducted in infants. The C-3 epimer was quantified in 97% of samples (mean = 25.6 ng/mL, *n* = 155), contributing to 36% of serum 25(OH)D, and the level decreased over the first year [32]. The levels of C-3 epimer were unrelated to gestational age, age after delivery, height, or weight, but a negative association was found with bilirubin and C-reactive protein. There is a strong suspicion that the fetus–placenta is a source of epimer production after the detection of epimers in maternal, cord, and fetus blood—a result occurring due to the utilization of advanced assays. 

## 5. Conclusions

Although the existence of various vitamin D forms (such as epimers) has been established, their clinical significance remains obscure. Furthermore, recent data show that at least one epimer form has activity in vitro. With the development of more advanced assays, a thorough understanding of the interplay among the various vitamin D forms can be achieved. Specific and accurate assays highlight that a considerable proportion of vitamin D exists as epimers. Currently, separate measurement of epimers, through advanced assays, could overcome significant analytical and clinical issues. Specifically, in the daily clinical setting, separate measurement of epimers could provide a useful tool for the accurate measurement of 25(OH)D during pregnancy, in order to explain some of the aspects of the current gap between observational and supplementation studies on the field. Further studies are required to establish additional in vivo roles of epimers on human pregnancy.

## References

[1] Muscogiuri G., Altieri B., Annweiler C., Balercia G., Pal H.B., Boucher B.J., Cannell J.J., Foresta C., Grübler M.R., Kotsa K. (2016). Vitamin D and chronic diseases: The current state of the art. Arch. Toxicol..

[2] Manson J.E., Brannon P.M., Rosen C.J., Taylor C.L. (2016). Vitamin D Deficiency—Is There Really a Pandemic?. N. Engl. J. Med..

[3] Zerofsky M.S., Jacoby B.N., Pedersen T.L., Stephensen C.B. (2016). Daily Cholecalciferol Supplementation during Pregnancy Alters Markers of Regulatory Immunity, Inflammation, and Clinical Outcomes in a Randomized Controlled Trial. J. Nutr..

[4] Sahoo S.K., Katam K.K., Das V., Agarwal A., Bhatia V. (2016). Maternal vitamin D supplementation in pregnancy and offspring outcomes: A double-blind randomized placebo-controlled trial. J. Bone Miner. Metab..

[5] Vaziri F., Dabbaghmanesh M.H., Samsami A., Nasiri S., Shirazi P.T. (2016). Vitamin D supplementation during pregnancy on infant anthropometric measurements and bone mass of mother-infant pairs: A randomized placebo clinical trial. Early Hum. Dev..

[6] Karras S.N., Fakhoury H., Muscogiuri G., Grant W.B., van den Ouweland J.M., Colao A.M., Kotsa K. (2016). Maternal vitamin D levels during pregnancy and neonatal health: Evidence to date and clinical implications. Ther. Adv. Musculoskelet. Dis..

[7] Grant C.C., Crane J., Mitchell E.A., Sinclair J., Stewart A., Milne T., Knight J., Gilchrist C., Camargo C.A. (2016). Vitamin D supplementation during pregnancy and infancy reduces aeroallergen sensitization: A randomized controlled trial. Allergy.

[8] Karras S., Anagnostis P., Naughton D., Annweiler C., Petroczi A., Goulis D. (2015). Vitamin D during pregnancy: Why observational studies suggest deficiency and interventional studies show no improvement in clinical outcomes? A narrative review. J. Endocrinol. Investig..

[9] Karras S.N., Anagnostis P., Bili E., Naughton D., Petroczi A., Papadopoulou F., Goulis D.G. (2004). Maternal vitamin D status in pregnancy and offspring bone development: The unmet needs of vitamin D era. Osteoporos. Int..

[10] Kovacs C.S. (2008). Vitamin D in pregnancy and lactation: Maternal, fetal, and neonatal outcomes from human and animal studies. Am. J. Clin. Nutr..

[11] Karras S.N., Shah I., Petroczi A., Goulis D.G., Bili H., Papadopoulou F., Harizopoulou V., Tarlatzis B.C., Naughton D.P. (2013). An observational study reveals that neonatal vitamin D is primarily determined by maternal contributions: Implications of a new assay on the roles of vitamin D forms. Nutr. J..

[12] Park H., Brannon P.M., West A.A., Yan J., Jiang X., Perry C.A., Malysheva O.V., Mehta S., Caudill M.A. (2016). Vitamin D Metabolism Varies among Women in Different Reproductive States Consuming the Same Intakes of Vitamin D and Related Nutrients. J. Nutr..

[13] Aghajafari F., Field C.J., Rabi D., Kaplan B.J., Maggiore J.A., O’Beirne M., Hanley D.A., Eliasziw M., Dewey D., Ross S. (2016). APrON Study Team. Plasma 3-Epi-25-Hydroxycholecalciferol Can Alter the Assessment of Vitamin D Status Using the Current Reference Ranges for Pregnant Women and Their Newborns. J. Nutr..

[14] Molnár F., Sigüeiro R., Sato Y., Araujo C., Schuster I., Antony P., Peluso J., Muller C., Mouriño A., Moras D. (2011). 1α,25(OH) 2–3-epi-vitamin D3, a natural physiological metabolite of vitamin D3: Its synthesis, biological activity and crystal structure with its receptor. PLoS ONE.

[15] Shah I., Petroczi A., Naughton D.P. (2012). Method for simultaneous analysis of eight analogues of vitamin D using liquid chromatography tandem mass spectrometry. Chem. Central. J..

[16] Shah I., Petroczi A., Naughton D.P. (2014). Exploring the role of vitamin D in type 1 diabetes, rheumatoid arthritis, and Alzheimer disease: New insights from accurate analysis of 10 forms. J. Clin. Endocrinol. Metab..

[17] Markestad T., Halvorsen S., Halvorsen K.S., Aksnes L., Aarskog D. (1984). Plasma concentrations of vitamin D metabolites before and during treatment of vitamin D deficiency rickets in children. Acta Paediatr. Scand..

[18] Kamao M., Tatematsu S., Hatakeyama S., Sakaki T., Sawada N., Inouye K., Ozono K., Kubodera N., Reddy G.S., Okano T. (2004). C-3 epimerization of vitamin D3 metabolites and further metabolism of C-3 epimers: 25-hydroxyvitamin D3 is metabolized to 3-epi-25-hydroxyvitamin D3 and subsequently metabolized through C-1alpha or C-24 hydroxylation. J. Biol. Chem..

[19] Singh R.J., Taylor R.L., Reddy G.S., Grebe S.K. (2006). C-3 epimers can account for a significant proportion of total circulating 25-hydroxyvitamin D in infants, complicating accurate measurement and interpretation of vitamin D status. J. Clin. Endocrinol. Metab..

[20] Shah I., James R., Barker J., Petroczi A., Naughton D.P. (2011). Misleading measures in vitamin D analysis: A novel LC-MS/MS assay to account for epimers and isobars. Nutr. J..

[21] Lutsey P.L., Eckfeldt J.H., Ogagarue E.R., Folsom A.R., Michos E.D., Gross M. (2015). The 25-hydroxyvitamin D3 C-3 epimer: Distribution, correlates, and reclassification of 25-hydroxyvitamin D status in the population-based Atherosclerosis Risk in Communities Study (ARIC). Clin. Chim. Acta.

[22] Lensmeyer G., Poquette M., Wiebe D., Binkley N. (2011). The C-3 epimer of 25-hydroxyvitamin D3 is present in adult serum. J. Clin. Endocrinol. Metab..

[23] Chailurkit L., Aekplakorn W., Ongphiphadhanakul B. (2015). Serum C3 epimer of 25-hydroxyvitamin D and its determinants in adults: A national health examination survey in Thais. Osteoporos. Int..

[24] Cashman K.D., Kinsella M., Walton J., Flynn A., Hayes A., Lucey A.J., Seamans K.M., Kiely M. (2014). The 3 epimer of 25-hydroxycholecalciferol is present in the circulation of the majority of adults in a nationally representative sample and has endogenous origins. J. Nutr..

[25] Djekic-Ivankovic M., Lavery P., Agellon S., Weiler H.A. The C-3α Epimer of 25-Hydroxycholecalciferol from Endogenous and Exogenous Sources Supports Normal Growth and Bone Mineral Density in Weanling Rats. http://jn.nutrition.org/content/early/2016/11/23/jn.116.231753.abstract.

[26] Tamblyn J.A., Hewison M., Wagner C.L., Bulmer J.N., Kilby M.D. (2015). Immunological role of vitamin D at the maternal-fetal interface. J. Endocrinol..

[27] Yazdanpanah M., Bailey D., Walsh W., Wan B., Adeli K. (2013). Analytical measurement of serum 25-OH-vitamin D3, 25-OH-vitamin D2 and their C3-epimers by LC–MS/MS in infant and pediatric specimens. Clin. Biochem..

[28] Hanson C., Anderson-Berry A., Lyden E., Kaufmann M., Wu A., Elliott E., Lee J.I., Jones G. (2015). Dynamics of Vitamin D Metabolism in Maternal-Fetal Dyads. J. Pediatr. Gastroenterol. Nutr..

[29] Carter G.D. (2012). 25-hydroxyvitamin D: A difficult analyte. Clin. Chem..

[30] Gallo S., Comeau K., Agellon S., Vanstone C., Sharma A., Jones G., L’abbé M., Khamessan A., Weiler H., Rodd C. (2014). Methodological issues in assessing plasma 25-hydroxyvitamin D concentration in newborn infants. Bone.

[31] Papapetrou P.D. (2010). The interrelationship of serum 1, 25-dihydroxyvitamin, D.; 25-hydroxyvitamin D and 24,25-dihydroxyvitamin D in pregnancy at term: A meta-analysis. Hormones (Athens).

[32] Granado-Lorencio F., Garcia-Heras L.M., Blanco-Navarro I., Pérez-Sacristán B. (2014). Assessment of 3-epi-25-OH-D3 in preterm and full term infant samples and its relationship to demographic, anthropometric and biochemical determinants. Clin. Biochem..

